# Perceived algorithmic control and gig workers’ work engagement: assessing the mediating role of psychological empowerment and the moderating effect of deep acting

**DOI:** 10.1186/s40359-025-03570-7

**Published:** 2025-11-07

**Authors:** Qingxiu Lin, Rui Sun, Qiuhua Zhu

**Affiliations:** 1 School of Business Administration, Huaqiao University, Quanzhou, Fujian 362000 China; 2 School of Management, Guangdong Ocean University, Zhanjiang, Guangdong 524000 China; 3 Oriental Enterprise Management Research Center of Huaqiao University, Fujian Humanities and Social Sciences Base, Quanzhou, Fujian 362021 China; 4 Quanzhou Normal University, Quanzhou, Fujian 362000 China

**Keywords:** Perceived algorithmic control, Work engagement, Psychological empowerment, Deep acting

## Abstract

**Background:**

Online labor platforms rely on algorithmic control to manage gig work, but its impact on work engagement remains contested. Existing research predominantly adopts technological determinism perspectives, neglecting gig workers’ agency, and lacking systematic exploration of motivational mechanisms and emotional resources. Based on Self-Determination Theory, this study examines how perceived algorithmic control influences work engagement through psychological empowerment, with deep acting as a moderator.

**Methods:**

Data were collected from Chinese gig workers (delivery riders/ride-hailing drivers, *N* = 392) through snowball and convenience sampling. Established scales measured core variables. Common method bias was tested using SPSS and AMOS, while PLS-SEM analyzed reliability, validity, and hypothesized pathways.

**Results:**

Perceived algorithmic control positively affects work engagement. Three psychological empowerment sub-dimensions—meaning, influence, and competence—partially mediate relationships between perceived algorithmic control sub-dimensions and work engagement respectively. Deep acting strengthens the positive effect of perceptual algorithm tracking evaluation on influence, and shows highest importance for work engagement but suboptimal performance. Among psychological empowerment sub-dimensions, meaning exhibits the most prominent importance and requires priority optimization.

**Conclusions:**

This study transcends technological determinism and validates the positive pathway through which algorithmic control enhances work engagement via psychological empowerment. It reveals meaning construction’s central role and deep acting’s differentiated moderating effects. Online labor platforms should optimize algorithm design, strengthen meaning perception, reduce ineffective monitoring, implement psychological empowerment incentive mechanisms, provide emotional resource support, and guide deep acting strategies.

**Supplementary Information:**

The online version contains supplementary material available at 10.1186/s40359-025-03570-7.

## Introduction

Online Labor Platforms (OLPs), which are customer demand-driven, rely on algorithmic control (AC) to achieve efficient supply-demand matching and intelligent labor processes management. This ensures gig workers maintain high efficiency and service quality. As a distinctive management mechanism, AC combines high control with high performance [1]. Its effects have attracted high attention from both academia and practice. Existing research presents two divergent perspectives on AC’s impact. The negative viewpoint suggests that gig workers, striving to maintain favorable digital reputations, must fully immerse themselves in role requirements throughout their work. This pressure may lead to overwork [2] and emotional exhaustion [3], making genuine work engagement challenging [4]. Conversely, the positive perspective posits that interactions with algorithms enable gig workers to establish emotional trust and commitment. This enhances cognitive work engagement [5]. Additionally, algorithmic technological support provides abundant work resources that facilitate high engagement for gig workers. Algorithmic fairness also contributes positively [6]. Empirical surveys have found that gig workers demonstrate high engagement levels [7]. These findings collectively indicate that AC’s effects are multifaceted.

Work engagement (WE) refers to a persistent positive, fulfilling affective-cognitive state that individuals exhibit in their work [8]. Research has shown that WE benefits gig workers on both individual [9] and organizational levels, particularly in enhancing work performance [10]. Examining AC’s impact on WE has significant implications for enhancing gig workers’ work experience and improving retention rates [11]. Service quality and customer experience remain fundamental to OLPs competition and survival [12]. Whether AC can ensure gig workers’ WE and service levels is crucial for testing of algorithmic management effectiveness. This holds critical importance for enhancing customer experience [13], establishing competitive advantages, and promoting sustainable OLPs development.

Existing research on the relationship between AC and gig workers’ WE has made significant progress, yet several limitations persist. First, existing studies predominantly examine this phenomenon solely from the job resources perspective [4, 5, 14]. This framework fails to adequately explain the paradoxical finding that gig workers maintain WE even when AC may lead to resource depletion [14]. Second, current research frequently employs technological determinism [15] to understand AC’s consequences. This approach overemphasizes technology’s agency and its deterministic impact on workers while neglecting gig workers’ subjective agency. Research indicates that interactive monitoring enhances cognitive engagement primarily by securing gig workers’ emotional trust and commitment [5]. This finding suggests the necessity of greater attention to gig workers’ subjective experiences and intrinsic motivational mechanisms.

Established research confirms that WE levels correlate closely with workers’ cognitive perceptions and attitudes toward their work [16]. There is increasing emphasis on employee subjectivity’s critical role in WE [17]. Notably, the on-demand work model of OLPs facilitates the cultivation of gig workers’ intrinsic motivation [18]. Algorithmic management further reinforces this intrinsic motivation through work design empowerment, promoting self-motivation among gig workers [19]. However, targeted empirical research providing evidentiary support for these theoretical propositions remains insufficient [20]. To address this gap, the present research employs self-determination theory as its theoretical foundation and introduces psychological empowerment (PE) as a mediating variable to elucidate the internal pathways through which AC influences gig workers’ WE.

Emotional labor (EL), as a widely adopted interpersonal interaction strategy in the service industry, constitutes a critical strategy for gig workers responding to AC [21]. Cross-industry research demonstrates that deep acting (DA) within EL enables individuals to adjust their internal emotional states to align with external expression requirements. This congruence between emotional experience and expression promotes proactive service behaviors [22]. It also replenishes emotional resources and mitigates occupational burnout [23]. Through DA strategies, gig workers systematically regulate and manage their emotions during the labor process. This essentially represents a self-investment in emotional capital. Such investment enhances individuals’ emotional capital reserves and alleviates negative emotional experiences [24]. It also facilitates smooth task completion through emotional provision [25]. Emotional resources constitute a necessary foundation for gig workers to maintain optimal working conditions [26]. Existing research primarily focuses on documenting the emotional challenges gig workers face in AC contexts [3, 27]. Discussions of coping strategies predominantly remain at the conceptual level [24]. Although emotional resources have received extensive attention as important moderating variables in traditional work contexts, their functional mechanisms within AC have not been adequately investigated. Based on these considerations, we incorporate DA as a moderating variable into our research framework. The investigation aims to thoroughly examine how gig workers’ emotional states influence their perceptions of PE and WE behaviors under AC environments. This approach provides novel theoretical perspectives and research directions for subsequent investigations.

Based on the preceding theoretical analysis, this study anchors itself in Self-Determination Theory to focus on gig workers’ intrinsic motivation. We construct a theoretical model with PE as the mediating variable and DA as the moderating variable. Through questionnaire survey methodology and PLS-SEM, the research examines the influence mechanisms of perceived algorithmic control (PAC) on gig workers’ WE. The investigation aims to address the following core research questions: (1) Does PAC affect gig workers’ WE, and if so, through what mechanisms? (2) Does PE function as a mediating variable in the relationship between PAC and WE? (3) How does DA moderate gig workers’ perceptions of PE and WE behaviors when confronted with AC?

The anticipated theoretical contributions are threefold. First, the research aims to validate the incentive efficacy of AC as a performance management system, thereby advancing the research agenda on AC. Second, it seeks to addresses an empirical gap by investigating AC’s empowerment effect on gig workers. The study explores the applicability of PE theory to emerging work modalities and provides a novel theoretical perspective for understanding gig workers’ WE. Third, it will examine the boundary conditions of AC mechanisms from an emotional resource perspective, proposing new conceptual pathways for future research.

At the practical level, the present research has important practical significance. From the OLPs governance perspective, the results provide substantive theoretical foundations for OLPs to optimize algorithmic management strategies. This optimization facilitates enhanced work experiences for gig workers, thereby increasing talent retention rates and fostering sustainable OLPs ecosystems development. From the individual practitioner standpoint, the conclusions offer strategic recommendations for emotional management among gig workers. These enable them to maintain optimal working conditions when confronted with AC mechanisms. The strategies enhance WE levels and service quality through systematic emotional resource management techniques.

## Theoretical foundation and research hypotheses

### Theoretical foundation

Self-Determination Theory (SDT) holds that individual behavioral drive, namely motivation, stems not only from external incentives but also from subjective desires. Human beings possess an innate desire for self-development and self-actualization, which leads to the formation of internal motivation. Internal motivation enables individuals to make autonomous behavioral choices, constituting the process of self-determination. When people work driven by internal motivation, their spontaneity, enthusiasm, and persistence become more pronounced [28]. In the context of information technology development and usage, SDT effectively explains the formation, transformation, and evolution of digital technology users’ behavioral motivations, providing a theoretical perspective for understanding human-computer interaction and other issues in the digital field [29].

PE is a form of intrinsic motivation that originates from a series of psychological cognitions formed by individuals in specific work environments. While organizational management practices play a crucial role in the empowerment process [30], the subjective perception and psychological experiences serve as the decisive factors in PE [31]. Extensive research have shown that individuals with high PE develop more positive perceptual experiences of work and exhibit more proactive work attitudes and behaviors [32]. Self-efficacy, work meaning, and related constructs have been shown to exert strong positive effects on WE [17, 33]. The significant promoting effect of PE on employee WE has been validated in various economies and occupational types [34, 35].

According to SDT, gig workers’ perceptions of AC reflect their willingness and perceived capability to engage in work. For example, when workers perceive algorithmic standardized guidance, it clarifies their work objectives; similarly, real-time algorithmic monitoring enables them to receive immediate feedback and adjust their action strategies accordingly. These processes may all be ways for gig workers to gain PE. Higher PE not only means that the self-efficacy of completing the task is more sufficient, but also the internal driving force of work is stronger. Consequently, when gig workers feel sufficiently empowered in their work, they are more likely to engage fully and approach work challenges with enthusiasm.

### Perceived algorithmic control and gig workers’ work engagement

AC is a control process through which OLPs use algorithms to monitor gig workers’ behavior [36] and ensure that it is consistent with the OLPS’ organizational objectives [37]. It is embedded within real-time monitoring processes, utilizing data collected through algorithmic monitoring systems to guide worker behaviors and provide performance feedback [38]. These data encompass not only worker behaviors but also digital feedback from customers [39]. AC thus facilitates multiple functions including algorithmic recommendation, algorithmic restriction, algorithmic recording, algorithmic rating, algorithmic replacement, and algorithmic reward—each reconfiguring the intrinsic logic of work characteristics to varying degrees yet with equally profound implications [15]. Broadly categorized, these functions form three major classifications: algorithmic restriction and algorithmic recommendation for worker guidance; algorithmic recording and algorithmic rating for worker evaluation; and algorithmic replacement and algorithmic reward for worker constraint [40]. PAC refers to gig workers’ perceptions and cognitions towards OLPs’ AC. Pei et al. (2021) conceptualized this construct along three dimensions: perceptual algorithm standardized guidance (PASG), perceptual algorithm tracking evaluation (PATE), and perceptual algorithm behavioral constraint (PABC) [12], corresponding respectively to the three functional dimensions of algorithmic control.

Algorithmic functionality creates a supportive work environment for gig workers [14]. On one hand, the de-skilling effect of algorithmic technology significantly reduces barriers to entry and operational complexity for gig workers [41]. Intelligent algorithms substantially enhance the efficiency of work-related computational processes within organizations, strengthening gig workers’ sense of competence. On the other hand, algorithms provide gig workers with a degree of autonomous choice, enabling them to accept tasks according to their personal preferences and capability levels. Whether through standardized guidance, tracking evaluation, or behavioral constraints, when gig workers perceive the various workplace resources provided by algorithms [17], they not only enhanced work flexibility and efficiency, but also satisfy basic psychological needs to some extent, thereby enhancing self-efficacy, stimulating work enthusiasm and vigor, ultimately promoting full engagement in work [42].

Algorithmic guidance primarily encompasses intelligent task allocation, standardized instructions, and real-time performance feedback. During the process of PASG, gig workers gain clear understanding of platform-established work standards and norms [43], receive substantial task-relevant informational support. These resources, on one hand, contribute to satisfying gig workers’ competence needs and enhancing their sense of work control [36]. On the other hand, clear work standards and timely feedback provide space for autonomous improvement and growth, facilitating the transformation of external work requirements into intrinsic motivation, thereby elevating WE. Based on these, his article proposes hypothesis:

- Hypothesis 1 Perceptual algorithm standardized guidance positively affects gig workers’ work engagement.

Algorithmic evaluation focuses on real-time monitoring of gig workers’ location, progress, and performance. In the process, gig workers benefit from the convenience of real-time access to and clear understanding the target progress and performance results [36]. Algorithmic monitoring and performance management help cultivate a sense of fairness [44], which has a facilitating effect on WE through challenging assessments [6]. Meanwhile, having timely feedback at work is an important job resource which possess inherent motivational characteristics that can stimulate motivation and enhance WE [42]. Moreover, this instant feedback mechanism enables gig workers to autonomously control their work pace and exert control over their work, which contributes to WE [42]. Hajiheydari and Delgosha (2024) empirically demonstrated that under algorithmic control, gig workers’ work autonomy and information sharing have significantly positive effects on their WE [45]. Therefore, this article proposes hypothesis:

- Hypothesis 2 Perceptual algorithm tracking evaluation positively affects gig workers’ work engagement.

Algorithmic constraint mainly ensure gig workers’ performance by setting a series of automatic rewards (cash bonuses, inclined dispatching, etc.) and penalties (such as account bans and monetary fines) [40]. First, algorithmic compensation as a job resource has a significantly positive impact on gig workers’ WE [45], and fair algorithmic performance management can challenge-promote WE [6]. Second, PABC links gig workers’ task status with task process-related information (such as customer requests, new orders, priority changes) and customer interfaces [46], while customer satisfaction ratings serve as key indicators for determining future tasks [47]. In this process, gig workers can clearly understand work requirements and behavioral outcomes, leading to enhanced service mindset [48], which in turn prompts them to proactively adjust their behavior to adapt to algorithmic logic. The incorporation of gamification elements is also a useful tool for ensuring gig workers’ WE [49]. Furthermore, many gig workers are passively “pushed into” this labor market for survival purposes [50], economic motivation is a determining factor for sustained WE [51]. Based on this, this article proposes hypothesis:

- Hypothesis 3 Perceptual algorithm behavioral constraint positively affects gig workers’ work engagement

### The mediating effect of psychological empowerment

PE represents a cognitive psychological process perceived by individuals, constituting an internal motivational structure comprising three dimensions: meaning, competence, and influence [52]. Extant research indicates that interactions between algorithmic human resource management systems exert differential effects on self-efficacy through technological stress [38]. This evidence demonstrates that various algorithmic system functions potentially generate distinct psychological experiences and perceptions among gig workers, suggesting researchers should deconstruct the internal mechanisms of PE from a micro-dimensional perspective.

Meaning refers to the congruence between personal values and work goals or objectives [52]. When employees feel that their work is meaningful and aligned with their personal value pursuits, they tend to show higher work enthusiasm and engagement [53]. PASG facilitates the construction of work meaning, which in turn promotes WE. Specifically: Through PASG, gig workers have clarified task goals and standard norms, and understood the meaning and value of their work. Extensive information sharing helps gig workers comprehend work processes and recognize their organizational roles and contributions. This organizational information sharing has been shown to enhance employees’ perception of work meaning [54]. Additionally, real-time performance feedback increases gig workers’ contact with work objectives, and high contact lead to enhanced perceptions of meaning [52]. Perceiving high degree of meaning is believed to bring about commitment, participation, and concentration of energy [53]. As gig workers come to understand the value and significance of their work, they become more willing to invest their time and energy. Based on this, this article proposes hypothesis:

- Hypothesis 4 Meaning mediates the relationship between perceptual algorithm standardized guidance and gig workers’ work engagement.
- Hypothesis a Perceptual algorithm standardized guidance positively affects meaning.
- Hypothesis b Meaning positively affects gig workers’ work engagement.

Influence is an individual’s belief that they can influence organizational outcomes and work decisions, including both the capacity and opportunity to exert influence. In service contexts, it reflects the extent to which employees can control customer’s service experiences (an organizational outcome) rather than just their own work activities (a self-determination outcome) [52]. PATE contributes to enhanced influence perception, which subsequently promotes WE. Algorithmic tracking evaluation provides real-time feedback about performance through continuous monitoring of work progress and attitudes. This information fundamentally provides evidence of personal behavioral control, thereby strengthening gig workers’ cognitive assessment of their own influence [53]. Positive monitoring and information feedback enhance gig workers’ perceptions, cognitions, and evaluations of their autonomy in labor processes [55]. When algorithms automatically evaluate work quality based on various monitoring metrics, both outcome fairness and procedural fairness directly affect employees’ perceived influence [56]. When gig workers perceive enhanced work influence, their proactivity and enthusiasm are activated, leading to increased intrinsic work motivation and, consequently, greater WE. Accordingly, this paper proposes hypothesis :

- Hypothesis 5 Influence mediates the relationship between perceptual algorithm tracking evaluation and gig workers’ work engagement.
- Hypothesis a Perceptual algorithm tracking evaluation positively affects influence.
- Hypothesis b Influence positively affects gig workers’ work engagement.

Competence, or self-efficacy, refers to individuals’ belief in their ability to successfully execute assigned tasks [52]. PABC are beneficial for enhancing competence perception, which in turn promotes WE. Algorithmic behavioral constraints implement a multi-faceted approach to performance management: they classify and rank work performance, distribute monetary rewards during specific periods, and impose penalties when work fails to meet requirements. Research has demonstrated that appropriately structured reward systems can significantly enhance employees’ perceived work competence [57]. Dermer (1975) [58] observed that employees who receive rewards tend to develop more optimistic and positive work attitudes, showing greater willingness to assume responsibilities and expend effort. If organizations provide reasonable, performance-based rewards, it will have an inherent motivating effect on employees, making them affirm their capabilities. The algorithmic incentive system serves a deeper psychological function: it signals the platform’s recognition and valuation of gig workers’ contributions and efforts, thereby strengthening their self-confidence. As individuals experience this reinforcement of their self-efficacy, their belief in their task completion capabilities strengthens. This enhanced self-belief subtly but significantly increases the likelihood of WE [31]. Accordingly, this article proposes research hypothesis :

- Hypothesis 6 Competence mediates the relationship between perceptual algorithm behavioral constraint and gig workers’ work engagement.
- Hypothesis a Perceptual algorithm behavioral constraint positively affects competence.
- Hypothesis b Competence positively affects gig workers’ work engagement.

### The moderating effect of deep acting

DA is a more positive form of EL, functioning as a resource replenishment process that effectively modifies workers’ internal emotional states. This emotional management strategy focuses primarily on inner emotional feelings [59]. Primarily, it operates as an antecedent-focused emotion regulation strategy, achieving emotional alignment with target states through situational or cognitive modifications [60]. During this process, individual cognition converges with work objectives, fulfilling autonomy needs and enhancing intrinsic work meaningfulness.

Furthermore, DA constitutes a crucial competency [26]. When gig workers actively engage in self-regulation during their work processes, first generating the “appropriate” emotions and subsequently manifesting required external behaviors driven by these emotions, they not only experience more positive affect but also enhance their sense of competence and self-efficacy. This proactive emotional regulation process also demonstrates autonomy, facilitating the internalization of external work requirements into self-identified behavioral standards.

Finally, DA facilitates affect delivery [61] and promotes authentic customer interactions, resulting in emotional and social relationship compensation [62]. This simultaneously satisfies relational needs while enhancing perceived influence. In essence, DA strengthens the PE induced by gig workers perceived algorithmic control. Accordingly, Hypotheses 7–9 are proposed as follows:

- Hypothesis 7 Deep acting positively moderates the relationship between perceptual algorithm standardized guidance and meaning.
- Hypothesis 8 Deep acting positively moderates the relationship between perceptual algorithm tracking evaluation and influence.
- Hypothesis 9 Deep acting positively moderates the relationship between perceptual algorithm behavioral constraint and competence.

**Fig. 1:**
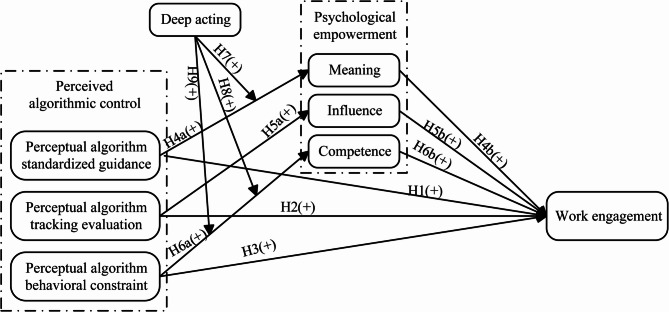
Theoretical framework

Based on the above research hypotheses, this article proposes a research framework as shown in Fig. 1.

## Materials and methods

### Research design

This study employed electronic questionnaires as the primary data collection method to gather data for statistical analysis and hypothesis testing. The main purpose of this study is to explore the mechanism and boundary conditions by which gig workers’ perception of algorithmic control affects their WE in labor processes controlled by algorithms. This category of algorithm-controlled, high-constraint, high-dependency programmatic matching gig work is concentrated in food delivery and ride-sharing industries [63], making the research subjects of this study very clear: delivery riders or ride-hailing drivers.

Due to the certain degree of autonomy in work time and location, the highly dispersed distribution, and the lack of a unified organizational framework or sampling frame, traditional random sampling methods are difficult to implement. This study primarily recruited participants through online snowball sampling and convenience sampling: (1) Initial respondents were randomly contacted online to complete the questionnaire, followed by chain-referral where they recommend other gig workers. (2) Gig workers were contacted to complete the questionnaire through the person in charge of the local rider station or the ride-hailing team leader. Each participant received monetary compensation as an incentive. The recruited participants were all individuals who are currently working as delivery riders or ride-hailing drivers, or have previous experience in such work, but were not limited by their work platform or employment nature (full-time/part-time). The G*Power 3.1.9.4 [64, 65] and rules of thumb for minimum sample sizes [66] were used to compute the necessary sample size before data analysis.

Prior to data collection, this study obtained ethical approval from the academic committee of the first author’s home university. The participants were verbal informed of the objective of this study prior to participating, if they agreed to join in this study voluntarily, they would receive a link to the questionnaire and proceed to complete it online. The beginning of the survey questionnaire explained the research objective and related information, as well as provided guidance for filling out the questionnaire. Participants completed the questionnaire as required, so it is also considered as giving informed consent, and that their anonymity was preserved.

To ensure measurement reliability and validity, all constructs were assessed using mature scales that have been tested for reliability and validity and widely used mature scales at home and abroad. PAC was measured using the scale developed by Pei et al. (2021) [12], which conceptualizes the construct along three dimensions: PASG (4 items), PATE (4 items), and PABC (3 items), totaling 11 items. WE was assessed using the scale developed by Schaufeli et al. [67], which consists of 9 items. For PE, the study utilized the 3-dimensional questionnaire originally developed by Spreitzer and modified by Fulford and Enz (1995) [52] for service environments. This scale encompasses three dimensions - meaning (3 items), competence (3 items), and influence (6 items)—with a total of 12 items. The measurement of DA used the 6-item scale validated by Rui and Song [22].

To ensure data quality and validity, particularly given the random nature of participant selection and the inability to conduct multi-stage surveys, the questionnaire incorporated three reverse-coded items. These items served as attention checks to identify and exclude unreliable responses, thereby enhancing data integrity. All items used a 5-point Likert scale, ranging from 1 (“Strongly Disagree”) to 5 (“Strongly Agree”).

For the collected sample data, common method bias was assessed using SPSS 27.0.1 and AMOS 24.0. After confirming the absence of severe common method bias, PLS-SEM was employed via SmartPLS4 for data analysis. PLS-SEM integrates advantages of principal component analysis and multiple regression analysis. It is founded on variance analysis and uses partial least squares methodology for model prediction and analysis, enabling simultaneous evaluation of both measurement (outer) and structural (inner) models [5]. PLS-SEM is particularly suitable for exploratory research, small-sample studies, or contexts with underdeveloped theoretical frameworks. Given this study’s focus on exploring the mechanism linking perceived algorithmic control to gig workers WE—where relationships between constructs remain at a nascent stage—PLS-SEM was deemed the appropriate analytical method for hypothesis testing.

### Sample collection

Of the 469 questionnaires distributed, 392 valid responses were retained after removing 77 invalid questionnaires based on criteria such as completion times under 120 s and inconsistent responses to reverse-coded items, with an effective response rate of 83.58%. Based-on Cohen (1992) criteria for medium effect size(d = 0.5), α = 0.05, and statistical power of 0.8, the minimum required sample size was 310. Our sample of 392 participants exceeded this threshold.

The demographic composition of the sample reflected the broader gig worker population, with food delivery riders accounting for 57.91% and ride-hailing drivers comprising 42.1% of respondents. Full-time gig workers represented 47.19% of the sample, while part-time workers made up 52.81%. The gender distribution showed a male majority at 79.59%, with female workers accounting for 20.41%. The age distribution centered primarily in the age of 26–35, representing 62.76% of respondents. Regarding work experience, 77.55% of participants had been engaged in gig work for less than three years, and 49.23% reported working more than six hours daily. These distributions align well with known characteristics of the gig worker population, supporting the sample’s representativeness.

Overall, these data distributions are basically consistent with news reports and information obtained through researchers’ field investigations, and are quite similar to the basic information of survey subjects in related research on Chinese gig workers [68, 69].

## Test and results

### Common method bias test

To address potential common method bias in the collected valid questionnaires, this study conducted Harman single factor test using SPSS 27.0.1. Unrotated factor analysis was performed on all measurement variables. The results showed that the explanatory power of the first common factor was 33.84%, less than 40% of the critical value. This indicates that there is no serious common method bias for the data [70], and the research conclusions will not be significantly affected.

Further adhering to Podsakoff et al.‘s (2003) suggests, controlling for the effects of a single unmeasured latent method factor to assess common method bias [70]. Following the theoretical framework, an eight-factor measurement model was constructed. A nine-factor model was subsequently specified by introducing a common method variance (CMV), and model fit indices between these two configurations were compared (Table 1). Results indicated that after adding the common method factor: (1) improvements in incremental fit indices (IFI = + 0.048, TLI = + 0.053, CFI = + 0.049) were below the 0.10 threshold; (2) reductions in absolute fit indices (RMR=−0.004, RMSEA=−0.018) did not exceed the 0.05 benchmark. Collectively, these results demonstrate no significant improvement in model fit, confirming the absence of substantial common method bias in our survey data.

**Table 1:** Assessment of common method bias using marker variable method

Model	χ^2^/df	IFI	TLI	CFI	RMR	RMSEA
8-factor model	2.020	0.918	0.909	0.917	0.039	0.051
8-factor model + CMV	1.425	0.966	0.962	0.966	0.035	0.033

### Reliability and validity test

Preliminary statistical analysis of the valid questionnaire data revealed several important characteristics of the distribution. The item-level data generally showed negative skewness, indicating a left-skewed distribution pattern. Additionally, the kurtosis values deviated from zero across items. These distributional characteristics indicate that the data do not follow a normal distribution, thus failing to meet the normality assumption required for CB-SEM. PLS-SEM demonstrates greater flexibility in handling non-normal data sets and provides more versatility in constructing latent variable relationships within the model [71]. As previously noted, PLS-SEM is particularly appropriate for this study due to its strength in exploratory research and theory development, capability in handling complex models, and robustness in prediction-oriented analysis. Given these considerations, PLS-SEM and SmartPLS4 were used to analyze the data.

Items PA21 from PATE and WE13 from WE were removed as their factor loadings below the recommended threshold of 0.708 [72]. After these adjustments, all latent variables’ Cronbach’s α exceeding the recommended threshold of 0.7, indicating good reliability. The CR values ranged between 0.70 and 0.95. These results, taken together, provide strong evidence for the internal consistency reliability of the measurement model [71, 73]. The convergent validity is determined by an AVE value greater than 0.50 and a factor loading greater than 0.7. As shown in Tables 2 and 3, the factor loadings, Cronbach’s α, CR values, AVE values, and discriminant validity values all met the statistical thresholds. These results collectively demonstrate strong convergent validity in the measurement model.

**Table 2:** Measurement model assessment: factor loadings, reliability and validity

Construct	Items	Outer loading	Cronbach’α	CR Rho_A	CR Rho_C	AVE
PASG	PA11	0.771	0.753	0.754	0.844	0.575
	PA12	0.762				
	PA13	0.727				
	PA14	0.772				
PATE	PA22	0.785	0.719	0.723	0.842	0.64
	PA23	0.795				
	PA24	0.82				
PABC	PA31	0.818	0.71	0.711	0.838	0.633
	PA32	0.78				
	PA33	0.788				
Meaning	PE11	0.889	0.823	0.833	0.894	0.739
	PE12	0.881				
	PE13	0.806				
Influence	PE21	0.742	0.847	0.85	0.887	0.566
	PE22	0.77				
	PE23	0.738				
	PE24	0.739				
	PE25	0.761				
	PE26	0.764				
Competence	PE31	0.831	0.839	0.841	0.903	0.757
	PE32	0.898				
	PE33	0.88				
WE	WE11	0.835	0.915	0.917	0.931	0.63
	WE12	0.799				
	WE21	0.842				
	WE22	0.834				
	WE23	0.772				
	WE31	0.803				
	WE32	0.712				
	WE33	0.742				
DA	DA11	0.824	0.921	0.921	0.938	0.716
	DA12	0.856				
	DA13	0.851				
	DA14	0.871				
	DA15	0.82				
	DA16	0.853				

**Table 3:** Discriminant validity of the measurement model

Construct	Number of Items	Discriminant Validity							
		PASG	PATE	PABC	Meaning	Influence	Competence	WE	DA
PASG	4	0.758							
PATE	3	0.621	0.8						
PABC	3	0.751	0.745	0.796					
Meaning	3	0.551	0.379	0.408	0.86				
Influence	6	0.3	0.343	0.396	0.524	0.752			
Competence	3	0.327	0.352	0.426	0.54	0.567	0.87		
WE	8	0.537	0.526	0.59	0.632	0.583	0.554	0.794	
DA	6	0.346	0.433	0.413	0.501	0.601	0.59	0.677	0.846

The items shown on the √AVE; off-diagonal elements are Pearson correlation estimates

### Hypothesis test

According to the analysis results, except for the measurement item DA14 for DA, which has an outer VIF value of 3.007, slightly higher than 3 but less than 5, the inner VIF and outer VIF of all measurement variables in the model are both less than 3, meeting the recommended criteria of inner VIF < 3 and outer VIF < 5. Therefore, there is no collinearity issue in the structural model [72].

The predictive power of the model was assessed through R^2^, a key indicator of model explanatory capability. The statistical results show that the R^2^ of Meaning, Influence, Competence, and WE are 0.298, 0.322, 0.302, and 0.504, respectively, indicating that the model has strong explanatory power, particularly notable for the primary dependent variable of WE, where the model explains over 50% of the variance [74].

The cross-validation redundancy test results showed that the Stone-Geisser’s Q² values for the endogenous latent variables were 0.276, 0.302, 0.28, and 0.445 for Meaning, Influence, Competence and WE, respectively, indicating that the model has good predictive correlation.

The SRMR of the model constructed in this study is 0.053, which is below the standard of 0.08 [75]. This indicates good model fit with the empirical data.

Collectively, these results—the acceptable VIF values, substantial R^2^ values, positive Q² statistics, and satisfactory SRMR—provide strong support for the structural model’s explanatory power and predictive relevance.

To test the proposed hypotheses, two key statistical analysis techniques PLS Algorithm and Bootstrapping in SmartPLS4 were used. These analyses focused on examining the direct path coefficients between latent variables to evaluate the hypothesized relationships. Specifically, the positive and negative relationships and strength of impacts between latent variables will be represented by standardized direct path coefficients, while *t*-values and *p*-values will be used to infer whether the path has statistical significance, thereby making the test results more reliable. The direct effects between latent variables are presented in Table 4.

**Table 4:** Structural model results: standardized path coefficients

Target Variable	Antecedent Variable	*R* ^2^	β	Ses	t	*p*	CI	Results
Meaning	H4a PASG	0.276	0.337	0.052	6.492	0***	0.233–0.435	Accepted
Influence	H5a PATE	0.302	0.106	0.044	2.398	0.017*	0.022–0.195	Accepted
Competence	H6a PABC	0.28	0.164	0.051	3.231	0.001**	0.065–0.261	Accepted
WE	H1 PASG	0.445	0.099	0.049	2.04	0.041*	0.004–0.197	Accepted
	H2 PATE		0.121	0.046	2.63	0.009**	0.029–0.209	Accepted
	H3 PABC		0.158	0.048	3.315	0.001**	0.062–0.25	Accepted
	H4b Meaning		0.257	0.044	5.802	0***	0.172–0.347	Accepted
	H5b Influence		0.225	0.044	5.094	0***	0.141–0.314	Accepted
	H6b Competence		0.152	0.044	3.434	0.001**	0.066–0.237	Accepted

*=*p* <.05

**=*p* <.01

***=*p* <.001 (two-tailed test)

The analysis revealed significant positive relationships across all predicted pathways, with all standardized path coefficients showing positive values and achieving statistical significance at the *p* <.05 level. Specifically, PASG (*β* = 0.099, *p* <.05), PATE (*β* = 0.121, *p* <.01), and PABC (*β* = 0.158, *p* <.01) all demonstrated significant positive effects on WE. Hypothesis , hypothesis and hypothesis are valid.

The test results of mediation and moderation effects are shown in Table 5. It can be seen several significant indirect effects. Meaning demonstrated a significant partial mediating effect between PASG and WE, with a mediation effect of 0.087 (*p* <.001), supporting Hypotheses 4, 4a, and 4b. The partial mediating effect value of influence between PATE and WE is 0.024, *p* <.05, supporting Hypotheses 5, 5a, and 5b. The partial mediating effect value of competence between PABC and WE is 0.025, *p* <.05, Hypotheses 6, 6a, and 6b are valid.

Regarding the moderation hypotheses, DA significantly strengthened the positive relationship between PATE and influence, the moderating effect value is 0.035, *p* <.01, supporting Hypothesis. However, DA did not significantly moderate either the relationship between PASG and meaning, or between PABC and competence. Consequently, Hypotheses 7 and 9 were not supported.

**Table 5:** Structural model results: mediating and moderating effects

	Relationships	β	Boot SE	t	*p*	Boot CI	Results
Total effect	PASG→WE	0.186	0.046	4.047	0***	0.097–0.276	-
	PATE→WE	0.145	0.046	3.153	0.002**	0.051–0.234	-
	PABC→WE	0.183	0.047	3.909	0***	0.089–0.274	-
Direct effect	H1 PASG→WE	0.099	0.049	2.04	0.041*	0.004–0.197	Accepted
	H2 PATE→WE	0.121	0.046	2.63	0.009**	0.029–0.209	Accepted
	H3 PABC→WE	0.158	0.048	3.315	0.001**	0.062–0.25	Accepted
Specific indirect effect	H4 PASG→Meaning→WE	0.087	0.02	4.256	0***	0.049–0.13	Accepted
	H5 PATE→Influence→WE	0.024	0.011	2.117	0.034*	0.005–0.048	Accepted
	H6 PABC→Competence→WE	0.025	0.01	2.398	0.017*	0.007–0.048	Accepted
	H7 DA x PASG→Meaning→WE	0.013	0.014	0.916	0.36	−0.014-0.041	No Sig
	H8 DA x PATE→Influence→WE	0.035	0.011	3.162	0.002**	0.015–0.057	Accepted
	H9 DA x PABC→Competence→WE	−0.01	0.009	1.079	0.281	−0.03-0.007	No Sig

*=*p* <.05

**=*p* <.01

***=*p* <.001 (two-tailed test)

### Importance-performance map analysis

In order to further explore the importance of PAC, PE and DA on WE and the performance of the survey samples, this study conducted an Importance-Performance Map Analysis (IPMA). This analytical approach helps identify key determinants that exhibit high importance but relatively lower performance, providing valuable insights for future management improvements [76]. According to the suggestion, this article first tests the outer weights of all variables, and the results show that the outer weights of all variables are positive, which meets the IPMA test criteria. The positive outer weights indicate that all indicators contribute meaningfully to their respective constructs, providing a solid foundation for the importance-performance comparison.

The IPMA results, presented in Table 6, reveal an intriguing pattern of relationships between the key variables and WE. DA emerged as the most important factor, demonstrating the highest importance with an effect value of 0.271, followed by meaning with an effect value of 0.257. Notably, PATE showed the lowest importance with an effect value of 0.145.

**Table 6:** Results of the importance-performance map analysis (IPMA)

Variable	Item	Importance	Performance	Variable	Item	Importance	Performance
PASG		0.186	74.231	Influence		0.225	62.445
	PA11	0.062	75.255		PE21	0.048	69.515
	PA12	0.062	74.298		PE22	0.056	67.092
	PA13	0.058	73.151		PE23	0.042	64.349
	PA14	0.063	74.107		PE24	0.05	57.972
PATE		0.145	76.188		PE25	0.05	60.204
	PA22	0.057	77.615		PE26	0.052	56.633
	PA23	0.059	75	Competence		0.152	74.51
	PA24	0.066	75.957		PE31	0.056	74.235
PABC		0.183	75.777		PE32	0.061	74.49
	PA31	0.079	75.51		PE33	0.058	74.809
	PA32	0.079	75.638	DA		0.271	68.98
	PA33	0.072	76.212		DA11	0.057	67.411
Meaning		0.257	67.068		DA12	0.051	72.258
	PE11	0.107	65.944		DA13	0.054	68.304
	PE12	0.104	63.903		DA14	0.054	68.814
	PE13	0.088	72.194		DA15	0.053	67.538
					DA16	0.05	69.707

When examining the performance, a different pattern emerges. PATE demonstrated the strongest performance at 76.188, followed closely by PABC at 75.777. In contrast, influence showed the lowest performance level at 62.445. This divergence between importance and performance scores suggests potential areas for targeted intervention.

A detailed item-level analysis revealed that item PE11 (“The gig work I do is very meaningful to me”) has the highest importance value of 0.107, yet its performance score of 65.944 indicates substantial room for improvement. This finding suggests that enhancing workers’ sense of meaning in their work could be a particularly effective lever for increasing WE.

Synthesizing these findings across the key constructs reveals several important insights. In terms of PAC, improvements in PASG appear more promising for enhancing WE compared to PATE or PABC. Within the PE, interventions targeting meaning show greater potential impact than those focused on influence or competence. While DA emerges as a crucial factor in promoting gig workers’ engagement, its mechanisms and potential applications warrant further investigation.

## Discussion

### Summary and interpretation of results

The analysis reveals significant direct positive relationships between all three dimensions of PAC and gig workers’ WE. Hypotheses 1, 2, and 3 are verified. This finding confirms that AC ensures a certain level of WE through constructing supportive work environments [14]. It demonstrates the effectiveness of algorithmic management strategies currently employed by OLPs. However, the IPMA results reveal performance deficiencies within the PASG and PABC dimensions. The most critical indicators require enhancement. Specifically, real-time performance feedback mechanisms may subject gig workers to implicit psychological pressure [19]. Algorithmic evaluation systems overly reliant on output metrics potentially engender perceptions of inequity and psychological detachment [37]. These findings suggest that despite AC demonstrating significant efficacy in enhancing WE, substantial optimization opportunities exist. Further refinement of management mechanisms is necessary.

PE partially mediates the relationship between PAC and gig workers’ WE. Meaning, influence, and competence partially mediating the relationship between PASG, PATE PABC, and WE, respectively. Hypotheses 4, 5, and 6 have been validated. Gig workers’ various perceptions of AC enhance their belief perception of meaning, influence, and competence in their work. This in turn promotes their WE. The IPMA results provide additional support for this interpretation. They show that the overall importance of each PE dimension in promoting gig workers’ WE is greater than that of each PAC dimension. It highlights that acknowledging and activating workers’ inherent agency and intrinsic motivation [28] can significantly enhance their positive work behaviors. The findings suggest that while AC provides the structural framework, PE serves as the crucial mechanism for translating this control into enhanced engagement.

The results highlight meaning as the most influential dimension of PE. It shows the highest importance effect value among the three dimensions in relation to WE. This reveals a fundamental insight into gig worker motivation. When gig workers perceive a fit between their personal values and organizational goals, they are more likely to demonstrate service-oriented behaviors. They prioritize customer experience and exhibit higher levels of WE. To put it more simply, when gig workers find genuine meaning in their work, both customer service quality and the overall work environment tend to improve [52]. Values aligned with goals are more important than self-efficacy. This aligns with Bucher, Fieseler, and Lutz’s (2019) [77] observation that individuals who perceive themselves and their work as “valuable” and “making a difference” demonstrate higher engagement in digital work contexts. Furthermore, this result resonates with a key principle in job crafting theory [78], which suggests that employees actively redesign their job to enhance their sense of meaning and engagement.

This relationship between meaning and WE has profound implications for understand gig work motivation. Rather than focusing solely on competence development or impact awareness, the results suggest an alternative approach. Helping workers connect their personal values with their work activities might be the most effective path to enhancing WE. This aligns with broader psychological theories about human motivation. Finding purpose and meaning often serves as a more powerful driver of sustained effort than mere skill mastery or perceived influence.

DA strengthens the positive relationship between PATE and influence. Hypothesis has been verified. However, DA did not significantly moderate the relationships between PASG and meaning, or between PABC and competence. This can be attributed to multiple factors.

First, examining labor market characteristics, many gig workers are passively “pushed” into this labor market for survival [46]. Compared to DA, which is complex and potentially resource-consuming [60], surface acting represents a more expedient short-term strategy [26]. It may be better aligned with gig workers’ practical needs. Additionally, the self-reported cross-sectional data collection method employed in the investigation may have certain limitations inaccurately reflecting DA practices.

Second, from the perspective of OLPs evaluation mechanisms, EL is intimately connected with gig workers’ ability to secure customer trust and positive reviews [79]. DA, by enhancing customer service experiences, can rapidly manifest in customer evaluation systems. High ratings potentially result in preferential task allocation. This algorithm-based feedback mechanism may reinforce gig workers’ cognitive association between DA and influence. It thereby produces significant moderating effects exclusively within this particular pathway.

Third, according to Resources Conservation Theory, the extent to which gig workers adjust their emotional states during service delivery is influenced by their psychological resource reserves. Due to generally low algorithmic transparency, gig workers may not be clear about when and what data is being collected during real-time location tracking and progress monitoring [80]. This comprehensive, opaque, and continuous algorithmic monitoring may generate anxiety, tension, or psychological resistance [81]. DA can supplement emotional resources internally. Therefore, its moderating effect is significant in PATE contexts. In contrast, the emotional resource consumption of gig workers under PASG and PABC remains relatively ambiguous. This results in DA’s moderating effect failing to manifest along these two pathways.

Fourth, cultural background factors cannot be overlooked. In collectivist cultural contexts, surface acting has relatively limited effects on emotional exhaustion and occupational burnout [82]. Concurrently, economic incentives embedded within PABC can effectively mitigate emotional dysregulation resulting from surface acting [83]. This cultural context may contribute to the comparatively lower prevalence and necessity of DA among Chinese gig workers, thereby constraining its moderating effects.

### Theoretical contributions

First, the present research empirically validates the positive effect of PAC on gig workers’ WE. It demonstrates the effectiveness of algorithmic management strategies. This finding supports the premise that high control facilitates high performance in platform-based work environments. OLPs employ sophisticated rating systems and algorithms to monitor and manage worker performance. They effectively create highly automated performance management system [1]. Through performance evaluation and incentives, OLPs can better manage and guide large numbers of distributed gig workers. They continuously optimize task allocation algorithms based on performance data, thereby enhancing overall operational efficiency. This research reveals that algorithms serve a more nuanced role than mere monitoring tools. They function as comprehensive support systems that provide resources through task allocation, service standards, and decision-making information to guide worker behavior. This perspective transcends traditional control-oriented views. It highlights the constructive role of algorithmic management in facilitating work processes and outcomes. By demonstrating how AC can create supportive work environments rather than just restrictive ones, the findings contribute to a more balanced understanding of algorithmic management in the platform economy.

Second, this study advances theoretical understanding by revealing the mechanisms through which PAC affects gig workers’ WE through PE, grounded in SDT. This perspective deepens understanding of how PE theory applies in digital work environments. While traditional research on PE has primarily focused on conventional employment relationships, the present research extends this theoretical framework into the context of algorithmic management. It demonstrates that PE plays a crucial mediating role between PAC and WE. Beyond validating the applicability of PE theory in new forms of work, it critically illuminates how AC can foster positive work behaviors by enhancing workers’ PE. This finding directly addresses Petriglieri et al.‘s (2018) [84] theoretical inquiry into achieving worker empowerment within the gig economy. Particularly noteworthy is the discovery that work meaning is more effective in stimulating WE than self-efficacy. This insight encourages scholars to pay greater attention to gig workers’ subjectivity and meaning construction. It suggests a shift in how they conceptualize motivation in algorithmically managed work environments.

Third, the present research provides an in-depth revelation of the multidimensional mechanism of PAC. Although empirical research often conceptualizes PAC as a first-order construct [85], theoretical discussions emphasize its rich connotations and diverse functions [15]. In response to this contradiction, this study constructs theoretical hypotheses at the sub-dimensional level through systematic literature review and theoretical analysis, subsequently validating them through empirical research. The findings not only reveal complex interactive mechanisms within technological governance contexts but also deepen accurate understanding of the PAC concept. They provide new theoretical perspectives for subsequent research.

Fourth, the investigation identifies and emphasizes the significance and complexity of DA within AC contexts. Primarily, it confirms that emotional resources exert a moderating function within the operational mechanisms of AC. This finding challenges traditional negative perspectives on algorithmic management. It proposes a more nuanced understanding—that the effectiveness of AC partially depends on emotional resources available to gig workers. Additionally, the findings concerning the differentiated effects of DA as an emotional resource across AC sub-dimensions respond, to some extent, to Dutli et al.‘s (2024) [86] call for in-depth examination of EL and emotional regulation within the gig economy. They provide novel theoretical insights for understanding the distinct operational mechanisms of various AC dimensions and their complex interactions with emotional resources.

Fifth, research indicates that PE may impact the selection of EL strategies [87], and DA can significantly impact WE [88]. Considering that communication technologies and processes of social reproduction have rendered EL processes among gig workers increasingly complex and alienated [89], the mechanism of the interaction between gig workers’ EL, PE, and WE has become further complex, which may be an important direction for future research.

### Practical implications

Based on the effectiveness of algorithmic management strategies, AC should not merely function as a monitoring mechanism. It should be designed as a supportive tool. OLPs can utilize algorithms to provide gig workers with clear task allocation, explicit service standards, and informational resources necessary for decision-making. This assists gig workers in better understanding work objectives and enhancing WE. Regarding performance management, the dynamic adjustment capabilities of AC should be fully leveraged. OLPs should continuously improve algorithmic models based on performance data, ensuring efficient resource allocation and consequently enhancing overall OLPs operational efficiency. During implementation, vigilance is necessary regarding potential algorithmic biases or inequities.

Based on the findings, OLPs can assist gig workers in identifying intrinsic motivations and constructing work meaning while implementing algorithmic management practices. OLPs should take into account gig workers’ occupational characteristics, then encourage them to develop and utilize occupational advantages according to their capabilities and interests. For instance, some food delivery riders have assisted in locating missing persons during their work, while others serve as mobile community grid managers. These exemplify successful cases of deriving work meaning and value through leveraging occupational characteristics. For gig workers who have clearly grasped their work meaning, OLPs should implement more flexible AC measures. This includes additional evaluation criteria and reward-punishment mechanisms.

Considering that gig workers and OLPs often lack long-term stable contractual and emotional connections in the platform economy, OLPs’ direct influence on gig workers’ work content may be relatively limited. This makes meaning-shaping more challenging than in traditional contexts. OLPs can draw inspiration from job crafting theory. For instance, by acknowledging the occupational positioning of flexible employment without overemphasizing internal career advancement systems, OLPs can return to the “transitional” nature of such occupations. This approach helps gig workers identify with their current work, understand work meaning, and develop more flexible and broader career perspectives. Certainly, within a collective cultural context, OLPs providing more direct and convenient channels for peer communication or experience-sharing zones represents a viable approach. It enhances occupational identification through establishing interpersonal connections.

Furthermore, gamification design can both ensure the implementation of original AC functions and enhance engagement through entertainment, challenge, and sense of achievement [90]. This benefits gig workers’ PE. OLPs should refine their work design approach by maximizing gamified interactions with gig workers. They should incorporate modules that provide intrinsic motivation within the gaming process. For instance, quantifying and visualizing workers’ experience progression can enhance their sense of control over work processes. It elevates PE and generate sustained work motivation.

Given the findings regarding DA, both OLPs and gig workers should acknowledge the inherent characteristics of the service industry. Under the inevitability of EL, it is crucial to facilitate gig workers’ cognitive restructuring. This promotes more DA while reducing surface acting. DA is an important ability, but it is not an easily learned skill [26]. For gig workers, DA necessitates modifying authentic feelings and cognitions. It requires adjusting internal psychological states to align with external emotional expressions. This “genuine” EL strategy requires substantial personal resource investment. OLPs should minimize unnecessary process monitoring, such as Meituan’s “Smile Campaign” (which randomly checks riders’ smiles through mobile phone cameras during delivery). Instead, OLPs should facilitate optimized utilization of limited emotional resources while providing support and replenishment for these resources. They could implement concepts like the “grievance bonus” found in traditional organizations, demonstrating organizational support and recognition. Additionally, OLPs should establish psychological support systems for gig workers. This includes providing essential counseling services while conducting emotional management training to help gig workers master DA techniques.

OLPs should cultivate a culture of respect, mutual trust, and genuine recognition. This involves treating gig workers as valued partners rather than disposable resources. By designing diverse organizational activities and engagement programs, OLPs can foster a sense of belonging and connection that transcends transactional work relationships. Long-term incentive mechanisms are crucial for sustaining worker engagement. OLPs should develop strategies that encourage stable, collaborative relationships with high-performing workers. By prioritizing gig workers’ psychological needs, OLPs can create more resilient, motivated, and committed gig work ecosystems that benefit both gig workers and the OLPs.

### Limitations and prospects

Like any empirical study, this research has several inherent limitations that provide opportunities for future scholarly investigation. First, the current study relies exclusively on self-reported data from gig workers, which potentially introduces bias through self-presentation effects. This approach may compromise the reliability of measurements, particularly for variables like WE, DA. Future research should consider methodological diversification, including in-depth interviews, longitudinal data collection, and scenario-based experiments to obtain more scientifically rigorous and accurate data. Second, the removal of two items (PA21 and WE13) from the established scales, while statistically justified to ensure the validity of our measurement model, may affect the direct comparability of our findings with those of prior studies that employed the original scales. Third, the current study did not comprehensively account for control variables. Previous research suggests that work characteristics such as employment type (full-time/part-time), work experience, and daily working hours potentially significantly affect gig workers’ WE. Subsequent research should integrate these factors to provide a more nuanced understanding of the investigated relationships.

The findings of this study also provide some implications for future research: First, the research highlighted the critical role of the “meaning” dimension in PE. This finding invites deeper exploration into organizational design strategies that can enhance work meaning, particularly within algorithmic management environments. Future researchers might investigate how to balance operational efficiency with meaningful work construction. Second, the complex interplay between EL and PE warrants further investigation. Researchers could develop a more comprehensive theoretical model integrating EL strategies (including deep and surface acting), PE, and WE. This approach would provide a more holistic understanding of their dynamic interactions. Third, the inconsistent moderating effects of DA suggest the need for more nuanced research. Future studies could explore how different types of algorithmic control interact with individual emotional regulation capabilities and subsequently impact work performance. This could unveil intricate mechanisms underlying worker adaptation in algorithmic management contexts.

## Conclusions

This study investigated the impact of PAC on WE among gig workers, utilizing PE as a mediating variable and DA as a moderating variable. Through a questionnaire survey of 392 gig workers, the research revealed several critical findings: PAC demonstrated significant positive direct effects on gig workers’ WE. PE played a crucial mediating role in the relationship between PAC and WE. DA significantly strengthened the positive effect of PATE on influence, but its moderating effect was not statistically significant in the relationships between PASG and meaning, or between PABC and competence. PE is an importance factor in promoting WE among gig workers. OLPs must recognize that technological efficiency alone is insufficient. To generate positive worker behaviors, platforms must simultaneously focus on internalizing PE alongside algorithmic technological improvements. The future research should further explore the relationship between EL and PE to provide a more comprehensive understanding of gig workers’ WE mechanisms.

## Supplementary Information

Supplementary Material 1: Questionnaire Items

Supplementary Material 2

## Data Availability

The raw data supporting the conclusions of this article will be made available by the authors, without undue reservation.

## Abbreviations

OLPs: Online labor platforms
AC: Algorithmic control
WE: Work engagement
PE: Psychological empowerment
PAC: Perceived algorithmic control
EL: Emotional labor
DA: Deep acting
SDT: Self-Determination Theory
PASG: Perceptual algorithm standardized guidance
PATE: Perceptual algorithm tracking evaluation
PABC: Perceptual algorithm behavioral constraint
CMV: Common method variance
IPMA: Importance-Performance Map Analysis
