# Supplementary material for: Perceived algorithmic control and gig workers’ work engagement: assessing the mediating role of psychological empowerment and the moderating effect of deep acting
Source: BMC Psychol. 2025 Nov 7;13:1237. doi: 10.1186/s40359-025-03570-7 (PMC12595896; doi:10.1186/s40359-025-03570-7)
Supplement: Supplementary file 1 — Supplementary Material 1: Questionnaire Items [file 40359_2025_3570_MOESM1_ESM.docx]

**Questionnaire Items**

| **Variable** | **Item** | **Content** |
| --- | --- | --- |
| Perceptual algorithm standardized guidance (PASG) | PA11 | The algorithm intelligently allocates my work tasks |
|  | PA12 | The algorithm provides normative instructions for my work based on platform standards |
|  | PA13 | The algorithm provides extensive information support related to completing work tasks |
|  | PA14 | The algorithm provides real-time dynamic feedback on work performance |
| Perceptual algorithm tracking evaluation (PATE) | PA21 | The algorithm tracks my geographical location in real-time |
|  | PA22 | The algorithm continuously tracks my work progress |
|  | PA23 | The algorithm monitors my work attitude in real-time |
|  | PA24 | The algorithm automatically evaluates the quality of my work completion |
| Perceptual algorithm behavioral constraint (PABC) | PA31 | The algorithm ranks me within the platform based on my work performance |
|  | PA32 | The algorithm provides cash rewards during specific periods to motivate me to work hard |
|  | PA33 | When my work fails to meet platform requirements, the algorithm imposes fines on me |
| Meaning (WM) | PE11 | The gig work I do is very meaningful to me |
|  | PE12 | My job activities are meaningful to me |
|  | PE13 | Gig work on platforms is important to me |
| Influence (WI) | PE21 | I can freely decide how to do my gig work |
|  | PE22 | I have a chance to exercise personal initiative in my gig work |
|  | PE23 | I decide on how to go about doing my gig work |
|  | PE24 | My opinion counts in decision making |
|  | PE25 | I have a great deal of control over my gig work |
|  | PE26 | I have influence over what happens in my gig work |
| Competence (WC) | PE31 | Gig work is well within my scope of my activities |
|  | PE32 | I am confident in my ability to do the gig work |
|  | PE33 | I have mastered the skills to complete gig work |
| Work engagement (WE) | WE11 | At my work, I feel bursting with energy |
|  | WE12 | At my job, I feel strong and vigorous |
|  | WE13 | When I get up in the morning, I feel like going to work |
|  | WE21 | I am enthusiastic about my gig work |
|  | WE22 | Gig work inspires me |
|  | WE23 | I am proud of the gig work that I do |
|  | WE31 | I am immersed in my work |
|  | WE32 | I get carried away when I am working |
|  | WE33 | I feel happy when I am working intensely |
| Deep acting (DA) | DA11 | When serving customers, I am not just outwardly enthusiastic, but also feel genuinely happy inside |
|  | DA12 | At work, I try to overcome negative emotions and serve customers with genuine warmth and friendliness |
|  | DA13 | Even when I know the customer is not right, I still empathize and solve their problem with sincerity and patience |
|  | DA14 | I attempt to feel the emotions and attitudes required by my work, rather than just pretending |
|  | DA15 | If I must display a specific emotion in front of customers, I will try to make it as genuine as possible, rather than faking it |
|  | DA16 | When I'm in a bad mood, I temporarily forget about the unpleasant for work needs, ensuring a positive attitude when facing customers |
