# Supplementary material for: Perceived algorithmic control and gig workers’ work engagement: assessing the mediating role of psychological empowerment and the moderating effect of deep acting
Source: BMC Psychol. 2025 Nov 7;13:1237. doi: 10.1186/s40359-025-03570-7 (PMC12595896; doi:10.1186/s40359-025-03570-7)
Supplement: Supplementary file 2 — Supplementary Material 2 [file 40359_2025_3570_MOESM2_ESM.pdf]

Huaqiao University Medical Ethics Review and Approval Form

Approval serial number (to be filled in by the committee)	Ethical research No. (M2023009)	Application Date	Apr. 19, 2023
**Project name**	Research on the cognitive neural mechanism of personalized recommendation user privacy coping	**Project start and end time**	Apr, 2023- Oct, 2024

**Project Category**	A☐. Human specimen collection B☐. Human specimen experiment C☐. New drug clinical trials D☐. Clinical trials of new devices E☐. New Technology Applications F☑. Others ((please specify) : event-related potential

Brief information about the applicant (project leader)

Name	Rui Sun	Gender	male	Education	Postgraduate
Office phone	13159010819	Mobile phone	13159010819	Email address	sunrui@hqu.edu.cn
Main Research Direction	Research on individual psychology and cognition				
Funding Sources	☐Government ☑Foundation ☐Company ☐International organization ☐Other				

Applicant (project leader) commitment:

I guarantee that all investigators involved in the research of this project will strictly follow the Charter of the Medical Ethics Committee of Huaqiao University, and consciously abide by the relevant principles, accept the supervision and inspection of the Medical Ethics Committee of Huaqiao University at any time, and voluntarily accept the punishment if there is any violation of the regulations.

Signature of the applicant (project leader): Sun rui Date : Apr. 19, 2023

Comments from the applicant's department (center) [if the director of the department (center) is the applicant, please sign the deputy director of the department (center)]:

I have reviewed this research project, the study design and methods and believe that they are all reasonable and that the researcher has sufficient funds to carry out the research. Therefore, the research department agrees to conduct this study and hope for receive further review from the Research Ethics Committee of Huaqiao University.

Signature of the director of the department (center): Lijunjie Date : Apr. 19. 2023

Ethics Committee Approval Opinion:

Following the review, the department agreed to proceed with the study.

Medical Ethics Committee of Huaqiao University (Signed) Chairman (signature): Tong Lei

Date : 2023. 5.16
